# Supplementary material for: SARS-CoV-2 is associated with changes in brain structure in UK Biobank
Source: Nature. 2022 Mar 7;604(7907):697–707. doi: 10.1038/s41586-022-04569-5 (PMC9046077; doi:10.1038/s41586-022-04569-5)
Supplement: Supplementary file 2 — Reporting Summary [file 41586_2022_4569_MOESM2_ESM.pdf]

nature portfolio

Corresponding author(s): Gwenaelle Douaud

Last updated by author(s): Feb 16, 2022

Reporting Summary

Nature Portfolio wishes to improve the reproducibility of the work that we publish. This form provides structure for consistency and transparency in reporting. For further information on Nature Portfolio policies, see our Editorial Policies and the Editorial Policy Checklist.

Statistics

For all statistical analyses, confirm that the following items are present in the figure legend, table legend, main text, or Methods section.

n/a	Confirmed	
☐	☒	The exact sample size (*n*) for each experimental group/condition, given as a discrete number and unit of measurement
☐	☒	A statement on whether measurements were taken from distinct samples or whether the same sample was measured repeatedly
☐	☒	The statistical test(s) used AND whether they are one- or two-sided<br>*Only common tests should be described solely by name; describe more complex techniques in the Methods section.*
☐	☒	A description of all covariates tested
☐	☒	A description of any assumptions or corrections, such as tests of normality and adjustment for multiple comparisons
☐	☒	A full description of the statistical parameters including central tendency (e.g. means) or other basic estimates (e.g. regression coefficient) AND variation (e.g. standard deviation) or associated estimates of uncertainty (e.g. confidence intervals)
☐	☒	For null hypothesis testing, the test statistic (e.g. *F*, *t*, *r*) with confidence intervals, effect sizes, degrees of freedom and *P* value noted<br>*Give P values as exact values whenever suitable.*
☒	☐	For Bayesian analysis, information on the choice of priors and Markov chain Monte Carlo settings
☒	☐	For hierarchical and complex designs, identification of the appropriate level for tests and full reporting of outcomes
☐	☒	Estimates of effect sizes (e.g. Cohen's *d*, Pearson's *r*), indicating how they were calculated

Our web collection on statistics for biologists contains articles on many of the points above.

Software and code

Policy information about availability of computer code

Data collection	Data were obtained from UK Biobank (available from UK Biobank upon data access application)
Data analysis	Data were analysed using the latest versions of FSL and FreeSurfer, and using MATLAB code supplied.

For manuscripts utilizing custom algorithms or software that are central to the research but not yet described in published literature, software must be made available to editors and reviewers. We strongly encourage code deposition in a community repository (e.g. GitHub). See the Nature Portfolio guidelines for submitting code & software for further information.

Data

Policy information about availability of data

All manuscripts must include a data availability statement. This statement should provide the following information, where applicable:

- Accession codes, unique identifiers, or web links for publicly available datasets
- A description of any restrictions on data availability
- For clinical datasets or third party data, please ensure that the statement adheres to our policy

All source data is available (upon data access application) from UK Biobank. See https://www.fmrib.ox.ac.uk/ukbiobank/covid/ for analysis code from this study.

Field-specific reporting

Please select the one below that is the best fit for your research. If you are not sure, read the appropriate sections before making your selection.

☒ Life sciences ☐ Behavioural & social sciences ☐ Ecological, evolutionary & environmental sciences

For a reference copy of the document with all sections, see nature.com/documents/nr-reporting-summary-flat.pdf

Life sciences study design

All studies must disclose on these points even when the disclosure is negative.

Sample size	Sample size was determined by data availability from UK Biobank. Exact samples sizes are listed in Methods.
Data exclusions	Extreme outlier values were removed on the basis of being more extreme than 8 times the median absolute deviation from the median for a given variable of interest (see Methods for full details).
Replication	N/A
Randomization	N/A
Blinding	N/A

Reporting for specific materials, systems and methods

We require information from authors about some types of materials, experimental systems and methods used in many studies. Here, indicate whether each material, system or method listed is relevant to your study. If you are not sure if a list item applies to your research, read the appropriate section before selecting a response.

Materials & experimental systems

n/a	Involved in the study	
☒	☐	Antibodies
☒	☐	Eukaryotic cell lines
☒	☐	Palaeontology and archaeology
☒	☐	Animals and other organisms
☐	☒	Human research participants
☐	☒	Clinical data
☒	☐	Dual use research of concern

Methods

n/a	Involved in the study	
☒	☐	ChIP-seq
☒	☐	Flow cytometry
☐	☒	MRI-based neuroimaging

Human research participants

Policy information about studies involving human research participants

Population characteristics	SARS-CoV-2 status based on primary care/hospital/public health records or two positive antibody lateral flow test positive results.
Recruitment	This study is part of the UK Biobank COVID-19 re-imaging project, which has imaging pre-pandemic for thousands of participants, and focused on inviting back for a second scan participants who had been infected with SARS-CoV-2, and matched controls (in terms of age, interval between scans, sex, ethnicity). Biases include those known for the UK Biobank, i.e. a generally wealthier, healthier, and less ethnically diverse population. Specific bias for the COVID-19 re-imaging project is that, because it is based on volunteering of previous UK Biobank participants, those who have been infected by SARS-CoV-2 tended to have milder symptoms (which can be seen as a strength rather than a weakness of this study).
Ethics oversight	Human subjects: UK Biobank has approval from the North West Multi-centre Research Ethics Committee (MREC) to obtain and disseminate data and samples from the participants (http://www. ukbiobank.ac.uk/ethics/), and these ethical regulations cover the work in this study. Written informed consent was obtained from all participants. A statement on this is included in the paper.

Note that full information on the approval of the study protocol must also be provided in the manuscript.

Clinical data

Policy information about clinical studies

All manuscripts should comply with the ICMJE guidelines for publication of clinical research and a completed CONSORT checklist must be included with all submissions.

Clinical trial registration	*Provide the trial registration number from ClinicalTrials.gov or an equivalent agency.*
Study protocol	*Note where the full trial protocol can be accessed OR if not available, explain why.*
Data collection	*Describe the settings and locales of data collection, noting the time periods of recruitment and data collection.*
Outcomes	*Describe how you pre-defined primary and secondary outcome measures and how you assessed these measures.*

Magnetic resonance imaging

Experimental design

Design type	UK Biobank brain imaging data resting-state functional scans
Design specifications	N/A
Behavioral performance measures	N/A

Acquisition

Imaging type(s)	UK Biobank brain imaging data: structural (T1, T2 fluid attenuation inversion recovery and susceptibility-weighted), diffusion, and resting-state functional scans. See Methods for full details.
Field strength	3T
Sequence & imaging parameters	Please see Miller et al., Nature Neuroscience 2016 for a full list of the imaging parameters.
Area of acquisition	Whole brain

Diffusion MRI ☒ Used ☐ Not used

Parameters	Diffusion data are acquired with two b-values (b = 1,000 and 2,000 s/mm2) at 2-mm spatial resolution, with multiband acceleration factor of 3 (three slices are acquired simultaneously instead of just one). For each diffusion-weighted shell, 50 distinct diffusion-encoding directions were acquired (covering 100 distinct directions over the two b-values). (No cardiac gating.)

Preprocessing

Preprocessing software	FSL 5.0.9, Freesurfer 6 and Freesurfer 7 for subcortical segmentations.
Normalization	Whenever applicable spatial normalisation was required (please see Miller et al., Nature Neuroscience 2016, and Alfaro-Almagro et al., Neuroimage 2018), non-linear registration was used based on the structural images (usually T1).
Normalization template	MNI152 and UK Biobank.
Noise and artifact removal	Please see full details in Miller et al., Nature Neuroscience 2016
Volume censoring	N/A

Statistical modeling & inference

Model type and settings	Regression/correlation (see Methods for full details).
Effect(s) tested	Effects of SARS-CoV-2 modulated by age (model based on a published meta-regression of 28 studies) - see Methods for details.

Specify type of analysis: ☐ Whole brain ☐ ROI-based ☒ Both

Anatomical location(s)	ROI ("imaging-derived phenotypes", IDPs) cover the entire brain, and the entire cortical surface for FreeSurfer generated ROIs. In addition, we have created some visualisations of the effects voxel-wise and vertex-wise.
Statistic type for inference (See Eklund et al. 2016)	N/A

Correction | All results are (at least) false-discovery rate (FDR) significant, and we also report their family-wise error (FWE) corrected p-values.

Models & analysis

n/a	Involved in the study
☐	☒ Functional and/or effective connectivity
☐	☐ Graph analysis
☐	☐ Multivariate modeling or predictive analysis

Functional and/or effective connectivity | Based on partial correlation

Graph analysis | *Report the dependent variable and connectivity measure, specifying weighted graph or binarized graph, subject- or group-level, and the global and/or node summaries used (e.g. clustering coefficient, efficiency, etc.).*

Multivariate modeling and predictive analysis | *Specify independent variables, features extraction and dimension reduction, model, training and evaluation metrics.*
